# Calm after the storm? Clinical phenotypes and risk markers in suicidal patients: data from a liaison-consultation psychiatric setting

**DOI:** 10.3389/fpsyt.2026.1787649

**Published:** 2026-02-26

**Authors:** Gianmarco Cinesi, Agnese Sciolto, Chiara Miriam Carioti, Francesca Di Maio, Elena Sofia Gaias, Francesca Scopetta, Filippo De Giorgi, Alfonso Tortorella, Giulia Menculini

**Affiliations:** 1 Section of Psychiatry, Department of Medicine and Surgery, University of Perugia, Perugia, Italy; 2 Division of Psychiatry, Department of Neuroscience and Sensory Organs, Hospital of Perugia, Perugia, Italy

**Keywords:** liaison-consultation psychiatry, general hospital, mood disorders, non-suicidal self-injury, suicide

## Abstract

**Introduction:**

Suicidality represents an increasing public health matter and is more frequently associated with higher psychiatric vulnerability and with greater severity of psychopathological manifestations. Suicide risk may arise from a complex interplay of socio-environmental and clinical factors, which can be promptly explored during psychiatric consultations following a suicide attempt (SA). The present study aims at clarifying the correlates of suicidality in a liaison-consultation setting, in order to characterize risk profiles that could be targeted by preventive strategies.

**Methods:**

The present analysis is based on data collected from psychiatric consultations conducted in medical settings at the University Hospital of Perugia. We used the Columbia Suicide Severity Rating Scale for the assessment of suicidal ideation (SI), SA, and non-suicidal self-injury (NSSI). Study participants were divided in those referred after a SA (SA group) and those referred for other psychiatric reasons (non-SA group), including SI and NSSI. Bivariate analyses were performed to assess significant differences between the groups and a logistic regression model using Firth’s penalized likelihood was created to evaluate the correlates of suicidality in our sample.

**Results:**

In our sample (N = 373, 61.1% females, mean age 47.99 ± 21.09 years), 129 patients (34.6%) were evaluated after a real, interrupted, or aborted SA. Patients in the SA subgroup were more frequently females (p=0.031) and younger (p<0.001), also reporting a higher prevalence of familiar psychiatric history (p=0.011), previous SA (p<0.001) and NSSI (p<0.001), life stressors in the past six months (p=0.044), and current DSM-5-TR diagnosis of depressive (p=0.028) and personality disorders (p<0.001). At the logistic regression model (p<0.001), the variables that resulted to be positively associated with SA were previous NSSI (OR = 5.92), previous SA (OR = 3.09), a diagnosis of depressive (OR = 2.65) personality disorder according to DSM-5-TR (OR = 2.36), life stressors during the past six months (OR = 1.88), whereas age was negatively associated with SA (OR = 0.97).

**Conclusions:**

Our findings underline the high prevalence of suicidality in liaison-consultation psychiatry and confirm that psychiatric consultation in the general hospital represents a crucial opportunity for comprehensive assessment of risk factors and early intervention. Younger individuals with a history of suicidal behaviors and NSSI should be considered a high-risk group and prioritized for targeted preventive strategies.

## 1 Introduction

Liaison-consultation psychiatry (LCP) represents a specialized domain of psychiatric practice that integrates mental health care into general medical settings (1). Psychiatrists working in this field provide expert clinical evaluations, diagnostic clarification, and treatment recommendations for patients with confirmed or suspected psychiatric conditions. These consultations, typically requested by other healthcare professionals, most commonly occur in inpatient medical wards or emergency settings. Within this framework, LCP holds a strategic position in general hospitals, frequently encountering patients admitted to medical units for stabilization following self-harm or suicide attempts (SA). This context offers a unique opportunity to identify and characterize individuals at high risk for suicidal behaviors, facilitating access to services, integrated care, and the implementation of early interventions that could interrupt the cycle of recurrent attempts (2). A core challenge in both research and clinical practice lies in accurately differentiating suicidal ideation (SI), SA, and non-suicidal self-injury (NSSI). This distinction carries critical implications for risk assessment, treatment planning, and legal considerations in hospital settings, where every decision can profoundly impact patient outcomes. Undeniably, suicide and suicidal behaviors represent major public health concerns with profound clinical, social, and economic implications that make suicide prevention a priority at the global level (3). The World Health Organization estimates over 700,000 annual suicide deaths worldwide, positioning suicide among the leading causes of death in young people and highlighting the urgency of prevention strategies across healthcare settings (4). The Global Burden of Disease study reported approximately 746,400 deaths and 33.5 million disability-adjusted life years due to self-harm—predominantly suicide—in 2021 (5). Suicide ranks as the third leading cause of death among individuals aged 15–29 years (6). In Italy, national surveillance data indicate thousands of annual suicide deaths, with persistent regional heterogeneity—such as elevated rates in northern regions—and temporal fluctuations in external-cause mortality (7, 8). Notably, non-fatal suicidal behaviors, particularly SA, far outnumber suicide completions, possibly serving as critical endpoints for secondary prevention, offering a window for timely psychiatric intervention (9). Risk factors for SA are multifactorial, spanning individual, clinical, sociodemographic, social, and environmental domains. At the individual level, psychiatric disorders—particularly major depressive disorder (MDD) and bipolar disorder (BD), schizophrenia-spectrum disorders, and substance use disorders—represent the strongest predictors (10, 11), alongside a history of prior attempts (12, 13) and NSSI (14). As for the latter, some evidence suggests NSSI may acutely reduce SI in the short term, functioning as a maladaptive coping mechanism for suicidal states, though it ultimately escalates long-term risk through habituation to self-injury (15). Additional clinical correlates of suicidality include comorbid anxiety disorders, SI, high impulsivity, aggression, psychological pain, and childhood adversity, which often interact synergistically. On the other hand, sociodemographic factors encompass younger age, female sex, unemployment, single/divorced/separated status, and lower educational attainment (16, 17). Social and environmental contributors include recent stressors, socioeconomic deprivation, social isolation, and exposure to suicide in family or community settings (17). Despite the crucial role of specific nosographic entities in defining higher suicide risk, diagnostic challenges in LCP must be acknowledged. Among these, the adequate and timely identification of mood disorders deserves specific attention, particularly concerning the elevated risk of misdiagnosis between MDD and BD. To note, BD carries an elevated suicidal burden, with risk amplified by younger age at onset, depressive predominant polarity, comorbid anxiety/substance use/cluster B personality disorders, and family history of suicide (10, 18). Patients labelled with recurrent treatment-resistant unipolar depression may represent unrecognized bipolar spectrum cases, especially when clinical information concerning illness course, mixed features, or family history are missing. Personality disorders also increase suicidal risk, particularly cluster B disorders, which are characterized by emotional dysregulation (19). Nevertheless, any personality disorder diagnosis heightens risk, compounded by substance use, insecure attachment, and childhood trauma, creating a complex interplay that demands holistic evaluation (20). In time-constrained LCP settings, identifying personality disorders remains challenging but essential for risk stratification and tailored discharge planning.

Despite the clinical salience of suicidal behaviors in general hospitals, data collected in Italian LCP settings concerning possible risk factors for suicidality are scant. This retrospective study aims to delineate sociodemographic, psychosocial, and clinical, correlates of SA among patients assessed by an LCP service in a tertiary general hospital, integrating the evaluation of self-injury history, diagnostic features, and psychosocial stressors in a real-world setting. Given the crucial role that LCP settings play in early detection and intervention, results from the present study may help enlightening a comprehensive risk profile to bolster community prevention efforts and reduce future attempts.

## 2 Materials and methods

### 2.1 Study design and setting

This study relied on a retrospective design. The study was conducted at the LCP service of the University Hospital of Perugia, which offers psychiatric consultations to all the medical and surgical inpatient units of the third-level hospital. Psychiatrists working in the service use a structured schedule for data collection, including sociodemographic data (age, biological sex, employment status), personal and familiar psychiatric history, medical history (including reason for the current hospitalization), ongoing treatments, reason for requesting the current consultation, and suggested interventions. For the present study, we reviewed all the psychiatric consultations performed in the time period July 2024–June 2025. In case of repeated psychiatric consultations during the hospitalization, we only extracted data concerning the first consultation for the present analysis.

### 2.2 Sample characteristics

In this retrospective study, we included only consultations with complete data; cases with missing information were < 5%, since the use of electronic medical records in the tertiary general hospital allows the thorough examination of socio-demographic and clinical variables even in case some information is missing during the single psychiatric evaluation. Patients included in the present analysis were ≥ 14 years old according to real-world clinical practice in our centre, since children and adolescents < 14 years old are usually evaluated in child and adolescent psychiatry settings. All patients (or their parents/legal representatives) signed an informed consent form for data treatment according to current data protection policies, provided in their medical records at the time of admission in inpatient units.

### 2.3 Clinical assessment and instruments

All the psychiatric diagnoses were made by senior psychiatrists working at the liaison-consultation service according to the Diagnostic and Statistical Manual of Mental Disorders, 5th edition, text revision (DSM-5-TR) criteria (21). The diagnostic assessment relied on comprehensive unstructured clinical interviews, review of medical records, and collateral information—e.g., history collection with relatives—when available. The assessment of suicidality was routinely performed by using the Italian version of the Columbia Suicide Severity Rating Scale (C-SSRS), an instrument that evaluates the full spectrum of suicidal ideation and behavior through structured questions. The scale explores the presence and intensity of SI as well as the occurrence of suicidal behaviors, defined as a self-injurious act committed with at least some intent to die (22). Senior psychiatrists working at the service administered the scale and received specific training for suicide evaluation according to the C-SSRS. Given the relevance of affective symptoms and their potential role in the occurrence of a SA, at our service we dedicate specific attention to the assessment of these symptom dimensions in this population. The evaluation of affective symptoms was performed by using validated instruments, particularly the Hamilton Depression Rating Scale (23), Hamilton Anxiety Rating Scale (24), and the Mania Rating Scale (25). We collected data based on the structured schedules that were used for patient evaluations in the routine clinical practice. We also integrated data concerning SI (including wish to die, nonspecific active thoughts, and active suicidal intent or plan), suicidal behaviors (including aborted or interrupted attempts, and actual SA), and NSSI as evaluated by the C-SSRS. The presence of clinically significant affective symptoms as evaluated by the scales used during the psychiatric consultations was considered as a dichotomic variable, using the following cut-offs: HAM-D ≥ 14 for depressive symptoms, HAM-A ≥ 18 for anxiety symptoms, and MRS ≥ 20 for manic symptoms (26). We adopted dichotomized cut-offs to reflect clinically meaningful symptom thresholds and to facilitate interpretability in an applied clinical context where communication with specialists in other disciplines is of utmost importance.

### 2.4 Statistical analysis

All the collected information was extracted into an anonymized electronic dataset created with IBM Social Package for Social Sciences (SPSS), v. 26. For data analysis we used R 4.5.1 software. We performed descriptive analyses to examine the distributional properties of the variables of interest in the sample. We used absolute frequencies and percentages for categorical variables. Continuous variables were considered as normally distributed according to the central limit theorem (27) and were described using mean as centrality measure and standard deviation as index of dispersion. The sample was divided into two subgroups according to the presence/absence of a current SA (SA/non-SA). The presence of a current SA was confirmed by the C-SSRS as for regular clinical practice. Given the internal procedure requiring a psychiatric consultation as soon as patients are conducted to medical wards after a self-injurious act, the sample was considered to be representative of all subjects conducted to our hospital after a SA. Group comparisons between SA and non-SA patients were carried out using bivariate analyses: categorical variables were examined with the Chi-square or Fisher’s exact test, whereas the Student’s t-test was used to compare continuous variables (p < 0.05). A binary logistic regression model with Firth’s penalized likelihood was used to examine the association between socio-demographic, clinical, and psychosocial variables with the outcome of interest. The dependent variable was a dichotomous indicator of current SA, evaluated with the C-SSRS. Independent variables entered into the model were socio-demographic and clinical variables that turned out to be significant at the bivariate analyses. We did not include in the model variables concerning the current psychiatric evaluation (e.g., current depressed mood), since this was performed after the SA. Given the relatively low number of outcome events and the presence of strong predictors, a bias reduced logistic regression based on Firth’s penalized likelihood was chosen to limit small-sample bias and to address potential issues of complete or quasi-complete separation. From a methodological standpoint, the use of Firth’s penalized logistic regression allowed us to obtain less biased estimates in the presence of strong predictors and relatively infrequent outcome events (28). Compared with conventional maximum likelihood logistic regression, Firth’s approach reduces small-sample bias and mitigates issues related to complete or quasi-complete separation, which are common in clinical datasets characterized by highly predictive variables. This strategy therefore enhances the robustness and interpretability of multivariable associations in a real-world setting. The model was fitted in R (version 4.5.1) using the logistf package, which implements Firth’s method for binary logistic regression. Penalized maximum likelihood estimates of regression coefficients, Wald-type or penalized profile likelihood confidence intervals, and p values were obtained for each predictor. Model significance was evaluated using the penalized likelihood ratio test, and results are presented as odds ratios (ORs) with 95% confidence intervals (95% CI) and corresponding p values.

## 3 Results

### 3.1 Sample description

In our sample (n=373), most patients were females (n=228, 61.1%) with a mean age of 47.99 ± 21.1. The majority of subjects were Italian (n=307, 82.3%), single (n=196, 52.5%), and unemployed (n=213, 57.1%). The most frequent reasons for psychiatric consultation were overall psychopathological assessment (n=117, 31.4%) and re-evaluation of psychopharmacological treatment (n=115, 30.8%). The prevalence of SA in the study population was 34.6% (n=129). The most frequent method among suicide attempters was medication overuse (n=89, 69%). As for psychiatric diagnoses according to DSM-5-TR, the highest prevalence was detected for depressive (n=84, 22.5%) and anxiety (n=76, 20.4%) disorders, followed by substance-related disorders (n=61, 16.4%) and BD (n=29, 7.8%). Only 4.8% (n=18) patients suffered from schizophrenia spectrum disorders. Notably, personality disorders were diagnosed in 23.3% (n=87) cases.

### 3.2 Correlates of suicidality in the sample

Patients in the SA subgroup were more frequently females (69% vs 57%, p=0.031) and displayed a significantly younger age (37.31 ± 20.36 vs 54.64 ± 19.24, p<0.001). We found that 23.3% patients in the SA group were of foreign nationality compared to 14% in the non-SA group (p=0.037), whereas there were no further differences in the remaining socio-demographic characteristics. The presence of psychiatric family history was more common in the SA group (24% vs. 13.1%; p=0.011) and patients who attempted suicide were more frequently being followed by community mental health services (50.4% vs 30.6%, p<0.001). Moreover, people belonging to this population received psychological support in a higher percentage of cases (30.2% vs 11.1%, p<0.001). As for psychopharmacological treatment, in the SA group we found mood stabilizers (31.8% vs 18.4%, p=0.005), and particularly lithium (53.7% vs 24.4%, p=0.010), being prescribed more frequently. A similar finding was highlighted for second-generation antipsychotics (SGAs) (34.9% vs 21.7%, p=0.009). Moreover, patients in this population were more likely advised to increase treatment doses during the week before the suicide attempt (10.9% vs 3.3%, p=0.006). Previous SA, evaluated by the C-SSRS, were significantly more frequent in the SA group (37.2% vs 4.9%, p<0.001), and so was NSSI (24% vs 1.2%, p<0.001). When analysing psychopathological correlates, in the SA group we found a higher prevalence of depressive (29.5% vs 18.9%, p=0.028) and personality disorders (42.6% vs. 13.1%; p<0.001). Life stressors in the preceding six months were more prevalent in the SA group (55.8% vs 44.3%, p=0.044). Patients in the SA group were more frequently assessed as having SI (31% vs. 2.9%, p<0.001). In this population, psychomotor retardation (16.3% vs 5.3%, p=0.001) and depressed mood (41.9% vs 20.5%, p<0.001) as evaluated by HAM-D were also more prevalent, while clinically significant anxiety assessed with HAM-A was less frequent (31.8% vs 44.3%, p=0.026). Recommendations for initiating or adjusting pharmacological therapy were more frequently observed in the SA group (55% vs 38%, p=0.017). In a higher percentage of cases, patients who attempted suicide were hospitalized in a psychiatric setting following the end of the medical observation (10.9% vs 0.4%, p<0.001). For complete bivariate comparisons see **Table 1**.

**Table 1:** Comparisons of sociodemographic and clinical variables between subjects who attempted suicide (SA) and those who did not (n=373).

	SA (n=129)	n-SA (n=244)	χ^2^/t	OR (95% C.I.)	p
Sociodemographic characteristics					
Female sex (n, %)	40 (69%)	105 (57%)	4.642	1.68 (1.07–2.64)	**0.031**
Age (mean, sd)	37.31 (20.36)	53.64 (19.24)	7.643	–	**<0.001**
Age < 18 years (n, %)	106 (17.8%)	235 (3.7%)	19.750	5.67 (0.54–12.66)	**<0.001**
Not Italian (n, %)	99 (23.3%)	34 (53.1%)	4.372	1.85 (1.07–3.20)	**0.037**
Living alone (n, %)	19 (14.8%)	51 (20.9%)	1.591	0.66 (0.37–1.18)	0.207
Unemployed (n, %)	76 (58.9%)	137 (56.15%)	2.239	0.61 (0.34–1.10)	0.135
Poor social network (n,%)	32 (24.8%)	58 (23.8%)	0.026	1.08 (0.65–1.78)	0.871
Clinical characteristics (n, %)					
Familiar psychiatric history	31 (24%)	32 (13.1%)	6.407	2.1 (1.21–3.63)	**0.011**
Previous psychiatric hospitalizations	30 (23.3%)	22 (9%)	19.163	3.85 (2.09–7.06)	**<0.001**
Psychiatric follow-up in CMH	65 (50.4%)	74 (30.6%)	13.260	2.31 (1.48–3.58)	**<0.001**
Psychological support	39 (30.2%)	27 (11.1%)	21.315	3.64 (2.09–6.31)	**<0.001**
Previous SA	48 (37.2%)	12 (4.9%)	62.817	11.46 (5.80–22.64)	**<0.001**
Previous NSSI	31 (24%)	3 (1.2%)	50.242	25.41 (7.59–85.06)	**<0.001**
Medical comorbidities	22 (17.2%)	143 (58.6%)	58.384	0.14 (0.08–0.24)	**<0.001**
Insomnia within 7 days prior hospitalization	14 (10.9%)	17 (7%)	1.201	1.63 (0.77–3.41)	0.273
Life stressors in the last six months	72 (55.8%)	108 (44.3%)	4.059	1.59 (1.03–2.44)	**0.044**
Current psychiatric comorbidities (n, %)	14 (25.0%)	32 (62.7%)	15.516	5.05 (2.20–11.58)	<0.001
Depressive disorders	38 (29.5%)	46 (18.9%)	4.848	1.80 (1.09–2.95)	**0.028**
Bipolar disorders	15 (11.6%)	14 (5.7%)	3.303	2.16 (1.01–4.63)	0.069
Schizophrenia spectrum disorders	2 (1.6%)	16 (6.6%)	3.581	0.22 (0.05–0.99)	0.058
Anxiety disorders	19 (14.7%)	57 (23.4%)	3.362	0.57 (0.32–1.00)	0.067
Eating disorders	11 (8.5%)	8 (3.3%)	3.784	2.75 (1.08–7.02)	0.052
Substance-related disorders	15 (11.6%)	46 (18.9%)	2.713	0.57 (0.30–1.06)	0.100
Personality disorders	55 (42.6%)	32 (13.1%)	39.487	4.92 (2.96–8.20)	**<0.001**
Current psychopharmacological treatment (n, %)					
Antidepressants	52 (40.3%)	73 (29.9%)	3.637	1.58 (1.01–2.47)	0.057
Mood stabilizers (any)	41 (31.8%)	45 (18.4%)	7.730	2.06 (1.26–3.37)	**0.005**
Lithium	22 (53.7%)	11 (24.4%)	6.557	3.58 (1.43–8.94)	**0.010**
Valproic acid	15 (36.6%)	21 (46.7%)	0.530	0.66 (0.28–1.56)	0.467
FGAs	12 (9.3%)	18 (7.4%)	0.000	1.07 (0.49–2.32)	1.000
SGAs	45 (34.9%)	53 (21.7%)	6.883	1.93 (1.20–3.10)	**0.009**
Benzodiazepines	63 (48.8%)	100 (41%)	3.080	1.64 (0.98–2.73)	0.080
Dosage increase within 7 days prior hospitalization	14 (10.9%)	8 (3.3%)	7.441	3.59 (1.46–8.80)	**0.006**
Current psychopathological features (n, %)					
Depressive symptoms (HAM-D ≥ 14)	54 (41.9%)	50 (20.5%)	18.115	2.79 (1.75–4.46)	**<0.001**
Manic symptoms (MRS ≥ 20)	3 (2.3%)	10 (4.1%)	0.349	0.56 (0.15–2.06)	0.554
Anxiety symptoms (HAM-A ≥ 18)	41 (31.8%)	108 (44.3%)	4.970	0.59 (0.37–0.92)	**0.026**
SI (C-SSRS)	40 (31%)	7 (2.9%)	60.879	16.27 (7.01–37.81)	**<0.001**

CMH, Community Mental Health service; C-SSRS, Columbia Suicide Severity Rating Scale; FGAs, First-generation antipsychotics; HAM-A, Hamilton Anxiety Rating Scale; HAM-D, Hamilton Depression Rating Scale; KMDRS, Koukopoulos Mixed Depression Rating Scale; MRS, Mania Rating Scale; NSSI, Non-suicidal self-injury; SA, Suicide Attempt; SI, Suicidal Ideation; SGAs, Second-generation antipsychotics.

Bold values indicate statistical significance (p<0.05).

**Figure 1:**
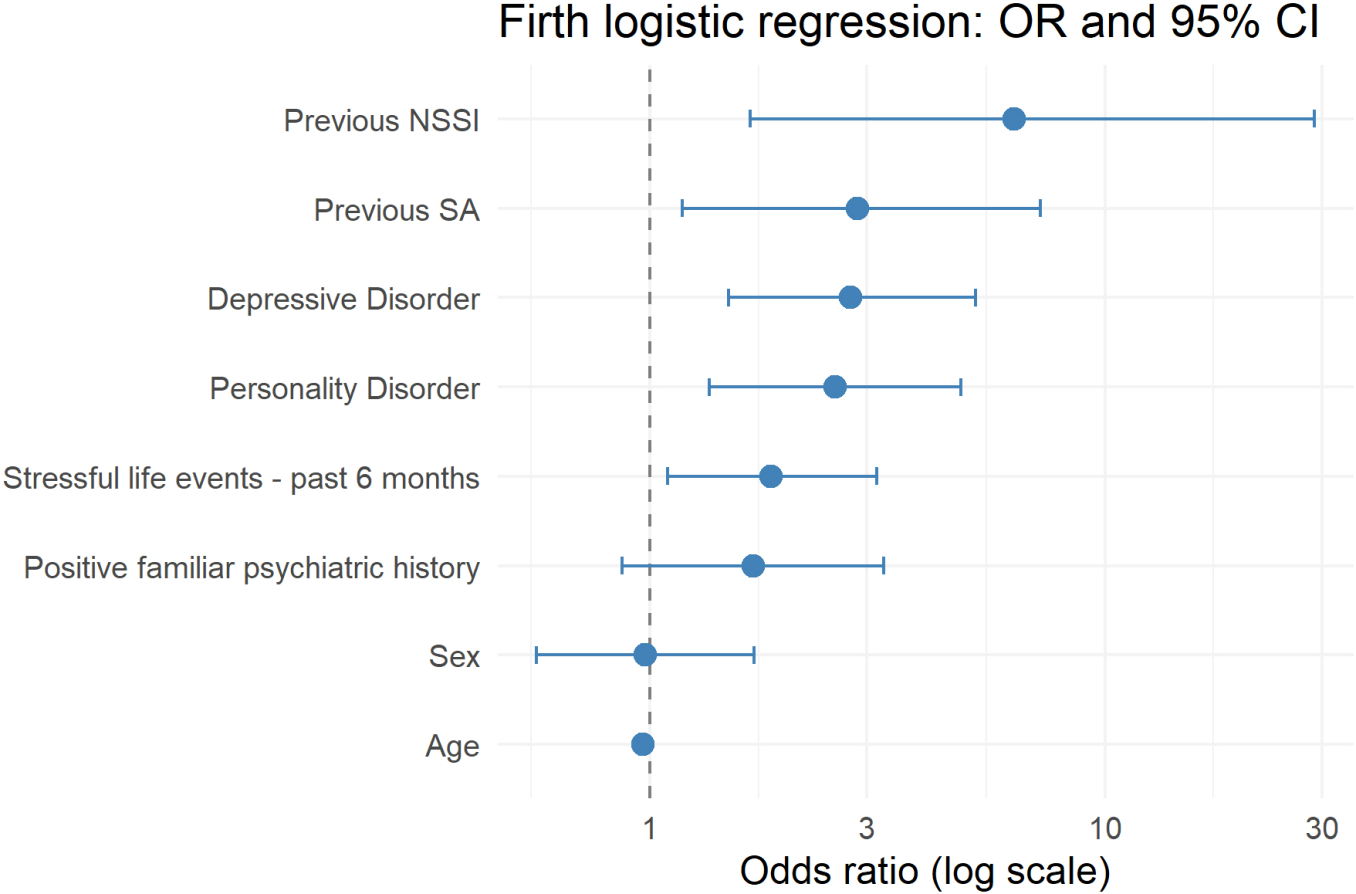
Strength of the associations between socio-demographic and clinical variables considered as covariates at the logistic regression and current suicidality (SA according to the C-SSRS).

A penalized binary logistic regression model using Firth’s bias-reduction method was fitted to account for potential small-sample bias and separation in the data. The model considered the presence of a current SA evaluated with the C-SSRS as dependent variable and included sex, age, psychiatric family history, previous SA, previous NSSI, depressive disorders, personality disorders, and stressful life events in the previous six months. The overall model was statistically significant (χ²(8) = 130.58, p < 0.001), suggesting good discriminative power. In the multivariable model, age was inversely associated with the presence of a current SA (OR 0.97; 95% CI 0.95–0.98; p < 0.001). Among the considered clinical variables, previous NSSI showed the strongest association (OR 6.31; 95% CI 1.66–28.86; p = 0.002), followed by previous SA (OR 2.86; 95% CI 1.18–7.21; p = 0.002). As for current psychiatric comorbidities, depressive disorders were associated with more than a twofold increase in the odds of the outcome (OR 2.76; 95% CI 1.49–5.20; p = 0.001), and PDs similarly increased the odds (OR 2.55; 95% CI 1.35–4.52; p = 0.004). The presence of stressful events in the previous 6 months was also significantly associated with higher odds of the outcome (OR 1.85; 95% CI 1.09–3.15; p = 0.02). A graphical representation of the main findings of the logistic regression can be found in **Figure 1**.

## 4 Discussion

In this LCP sample, SA accounted for more than one third of all psychiatric referrals from medical wards, confirming suicidality as a highly prevalent and clinically relevant condition in the general hospital setting. This is consistent with previous findings, also confirming high suicidal risk in LCP setting (29) and suggesting that suicidality should routinely be investigated in the general hospital, not depending from the reason of admission. Beyond prevalence, our findings contribute to a more refined understanding of individual and clinical profiles of patients evaluated after a SA, identifying a limited set of robust clinical and psychosocial correlates that retain significance in the multivariable analysis. The strongest associations with a current SA were observed for previous NSSI and previous SA, both remaining highly predictive after adjustment for psychiatric diagnoses and recent stressors, in line with previous findings (12–14). This pattern supports the conceptualization of suicidal behavior as a longitudinal and dynamic process rather than an isolated event, in which repeated exposure to self-injury progressively lowers the threshold for subsequent suicidal acts. In this framework, NSSI emerges not merely as a comorbid behavior, but as a marker of enduring vulnerability, possibly related to impaired emotion regulation, behavioral disinhibition, and habituation to self-inflicted harm (30). The magnitude of this association in a medically hospitalized population underscores the clinical relevance of systematically assessing lifetime NSSI during liaison-consultation evaluations, even when the referral reason is not primarily psychiatric. Current depressive disorders and personality disorders were also independently associated with SA, as expected on the basis of previous findings in LCP settings (10, 19, 20). While the association between depressive features and suicidality is well established (31), our findings suggest that diagnostic categories retain predictive value even when proximal affective symptoms are excluded from the regression model. This supports a dimensional interpretation in which depressive disorders reflect broader vulnerability traits, including hopelessness, cognitive rigidity, and reduced stress tolerance. It should also be noted that more than a half of SA patients were assessed as euthymic during the psychiatric consultation. This finding support a dynamic framework for the acute suicide crisis, challenging the traditional opinion that suicidality arises from persistent depressive states. Indeed, some individuals may exhibit a decline in depressive symptoms in the weeks following a SA (32). The transient reduction in subjective distress following a SA may be the resultant of complex and potentially intertwined phenomena, e.g., emotional exhaustion, temporary relief after externalizing unbearable psychological pain, or the perception that the crisis has been momentarily resolved. However, this apparent “calm after the storm” may be misleading, since suicidal behavior should be conceptualized as a dynamic and longitudinal process rather than a discrete event, in which acute post-attempt calm may coexist with persistent vulnerability and elevated risk of recurrence. In this perspective, the association with personality disorders highlights the contribution of enduring psychopathological features—affective instability, impulsivity, and interpersonal dysregulation, as specifically seen in cluster B personality disorders (33) and people with high neuroticism (34)—that may amplify the impact of acute stressors and medical illness. In LCP settings, where diagnostic assessment is often constrained by time pressure and overall clinical complexity, the identification of personality characteristics may be particularly challenging but appears clinically meaningful for suicide risk stratification. An additional, non-mutually exclusive interpretative hypothesis is that part of the observed association between depressive and personality disorders and SA may reflect the presence of bipolar spectrum conditions that are not fully recognized at the time of assessment. In real-world clinical practice, both in acute hospital settings and in community mental health services, diagnostic formulations may legitimately prioritize symptom management and functional stabilization, while longitudinal features such as mood periodicity, polarity shifts, and lifetime course may receive less systematic attention. Within this framework, recurrent or treatment-resistant MDD diagnosis may, in some cases, represent provisional or pragmatic formulations applied to patients with underlying mood instability. Although this hypothesis cannot be directly tested within the present study, it underscores the importance of a longitudinal, course-based diagnostic approach in LCP, particularly in patients presenting after suicidal behavior. This should be empowered by the systematic evaluation of specific “red flags” that should be considered among risk factors for a bipolar diathesis, e.g., familiar history of BD and early onset (35, 36), which also turned out to be significant in our analysis. The presence of stressful life events in the six months preceding hospitalization emerged as an additional independent correlate of SA. This finding aligns with stress-diathesis models of suicidality, in which environmental stressors interact with pre-existing vulnerability to precipitate suicidal behavior. Importantly, stressful events retained significance after controlling for psychiatric diagnoses and prior suicidal behaviors, supporting their role as proximal triggers rather than epiphenomena. From a clinical standpoint, this reinforces the importance of integrating psychosocial assessment into LCP practice, rather than limiting evaluations to symptom-based screening or diagnostic labels (37). Moreover, the importance of life adversities in the suicidal crisis suggests that a thorough screening of these events should be performed with special interest to specific vulnerable groups, that are both prone to suicidality and often experience highly stressful events (38). Age also showed a significant association with suicidality, with younger individuals displaying a higher likelihood of SA. This result is consistent with epidemiological data indicating higher rates of SA and non-fatal suicidal behavior among younger patients (39, 40) and suggests that age-related factors may be particularly relevant in hospital-based populations. Several developmental and contextual determinants may contribute to this pattern, including greater impulsivity, emotional reactivity, and exposure to acute interpersonal stressors during adolescence and young adulthood. Moreover, this life stage is characterized by identity instability and ongoing psychosocial transitions, which may amplify vulnerability to suicidal crises (41, 42). Contemporary stressors, such as social media-related pressures, peer comparison, and cybervictimization, have also been linked to increased suicide risk in younger populations. Conversely, older adults may be underrepresented in hospital-based liaison-consultation samples because suicidal behavior in later life is more likely to result in medically lethal outcomes, thereby reducing the probability of subsequent psychiatric evaluation (17). Within this framework, the observed age effect likely reflects both developmental vulnerability and selection mechanisms intrinsic to general hospital settings. These findings support the need for age-sensitive preventive strategies, with particular attention to early identification and intervention in younger patients presenting with self-injurious behaviors. Although female sex was associated with SA at the bivariate level, it did not retain significance in the multivariable model, indicating that sex differences may be largely mediated by clinical and psychosocial variables rather than representing an independent risk factor in this context. Beyond variables that remained significant in the multivariable model, several additional factors showed significant differences between SA and non-SA patients at the bivariate level. This pattern suggests that these features may primarily operate as indicators of overall clinical severity and of more intensive prior contact with specialist services, rather than as specific and independent risk markers for SA in the acute hospital setting. Foreign nationality also emerged as significantly associated with SA at the bivariate level. This finding warrants clinical consideration, as migration-related factors may meaningfully shape vulnerability to suicidal behavior (43). Indeed, individuals with a migrant background often face cumulative psychosocial stressors, including language barriers, limited access to healthcare, cultural incongruence in help-seeking behaviors, and experiences of marginalization (44). Acculturative stress—defined as the psychological burden associated with adapting to a new sociocultural environment—has been consistently linked to increased depressive symptoms, psychological distress, and suicidal ideation, particularly in recently migrated or socially isolated individuals (45). Reduced mental health literacy and communication difficulties may hinder early detection of affective symptoms, leading to delayed referral and presentation at more advanced stages of crisis in general hospital contexts (46). From a clinical perspective, this may underscore the importance of culturally sensitive assessment in LCP, with systematic attention to language proficiency, migration history, social support, and acculturative difficulties. Integrating these dimensions into routine risk assessment may improve the understanding of suicidal crises and facilitate more tailored discharge planning and continuity of care. In line with previous work showing that the apparent impact of sociodemographic and treatment-related variables often attenuates after controlling for psychiatric disorders and prior suicidal behaviors, our findings support the notion that the core vulnerability is carried by lifetime suicidal trajectories and underlying psychopathology, while other bivariate correlates may function as proxies of such vulnerability or markers of clinical selection into care. From a clinical perspective, the higher intensity and complexity of pharmacological regimens and psychosocial interventions in the SA group likely mirror histories of recurrent crises and persistent risk, rather than implying a direct causal role of specific medications or therapeutic settings in precipitating attempts. Overall, these results support a model of suicidality in which LCP serves not only as an interface service, but as a critical clinical setting for identifying enduring vulnerability markers that may otherwise remain unrecognized in medically ill patients. Indeed, the post-attempt phase represents a clinically critical window, during which reduced overt distress and a state of apparent “calm” may often obscure persistent drivers of suicidality.

### 4.1 Limitations

Several limitations should be acknowledged in the present study. The observational design precludes causal inference, and the single-center nature of the study may limit generalizability of findings. The relatively small sample size might have also influenced the limited number of cases observed in some diagnostic groups, e.g., patients suffering from schizophrenia spectrum disorders, which did not turn out to be significant at the bivariate analyses. Moreover, psychiatric diagnoses were based on clinical assessment, due to the real-world nature of the study that did not allow to administer structured diagnostic interviews, including those designed for the evaluation of personality disorders. However, this also represents a strength, as the findings reflect real-world liaison-consultation practice and variables that are readily accessible during routine hospital evaluations. Similarly, the evaluation of suicidality with the C-SSRS was performed in absence of formal inter-rater reliability assessment, reflecting the real-world nature of the service where the study was conducted.

## 5 Conclusion

Suicidality represents a frequent and clinically complex presentation in liaison-consultation psychiatry. In this general hospital sample, suicide attempts were most strongly associated with prior self-injurious behaviors, depressive and personality disorders, recent stressful life events, and younger age, rather than with acute symptom severity alone. These findings highlight the importance of a longitudinal and multidimensional approach to suicide risk assessment during psychiatric consultations in medical wards, with systematic attention to lifetime suicidal behaviors and NSSI. In this context, integrating course-based diagnostic perspectives may be particularly relevant to avoid oversimplified formulations centered exclusively on current symptom states, especially in patients presenting after suicidal behavior. Liaison-consultation psychiatry offers a unique opportunity to intercept high-risk individuals at a critical point of contact with healthcare services, particularly those who are not engaged in ongoing psychiatric care. Early identification of patients with established vulnerability profiles should be prioritized for targeted preventive strategies and structured follow-up pathways. Future longitudinal studies are warranted to determine whether interventions initiated during medical hospitalization can effectively modify long-term trajectories of suicide risk and reduce recurrence.

## Data Availability

The raw data supporting the conclusions of this article will be made available by the authors, without undue reservation.
